# Ethics of pad in mental disorders

**DOI:** 10.1192/j.eurpsy.2021.116

**Published:** 2021-08-13

**Authors:** M. Trachsel, R. Jox

**Affiliations:** 1 Clinical Ethics Unit, University Hospital of Basel (USB), Basel, Switzerland; 2 Clinical Ethics Unit, University Psychiatric Clinics (UPK) Basel, Basel, Switzerland; 3 Institute Of Biomedical Ethics And History Of Medicine, University of Zurich (UZH), Zurich, Switzerland; 4 Institute Of Humanities In Medicine, Lausanne University Hospital, Lausanne, Switzerland

**Keywords:** physician assistance in dying, decision-making capacity, ethics, Mental illness

## Abstract

Involuntary psychiatric hospitalization for suicide prevention and physician assistance in dying (PAD) for patients with severe and persistent mental illness (SPMI) combine to create a moral tension. Switzerland has the longest history of non-medicalized assistance in dying, considered as a civil right even beyond pathological situations. The debate in Switzerland centers on the notion of suffering in the context of PAD. In 2018, the Swiss Academy of Medical Sciences revised their end-of-life policy stipulating intolerable suffering due to severe illness or functional limitations and acknowledged as such by the physician as a core criterion for PAD. However, we argue that suffering is a necessary but insufficient condition for PAD, the other criteria being decision-making capacity (DMC) and refractoriness of the suffering. Moreover, we hold that suffering is a subjective experience that can only be quantified by the patient and cannot be compared between two persons in an objective way. According to this concept, however, some patients with SPMI, refractory suffering, and preserved DMC will meet the criteria for PAD. Therefore, we call for palliative care approaches in psychiatry which includes relief of suffering as much as possible, but also accepting PAD after a conscientious assessment of the criteria.

**Disclosure:**

No significant relationships.

